# PPARs, Cardiovascular Metabolism, and Function: Near- or Far-from-Equilibrium Pathways

**DOI:** 10.1155/2010/783273

**Published:** 2010-07-27

**Authors:** Yves Lecarpentier, Victor Claes, Jean-Louis Hébert

**Affiliations:** ^1^Service de Physiologie, Hôpital de Bicêtre, Assistance Publique-Hôpitaux de Paris, 94275 Le Kremlin-Bicêtre, France; ^2^Département de Physiologie Humaine, Université Paris 11 Sud, 94275, Le Kremlin-Bicêtre, France; ^3^Centre de Recherche Clinique, Centre Hospitalier Régional de Meaux, 77100, Meaux, France; ^4^Department of Pharmaceutical Sciences, University of Antwerpen, 2670, Wilrijk, Belgium

## Abstract

Peroxisome proliferator-activated receptors (PPAR *α*, *β*/*δ* and *γ*) play a key role in metabolic regulatory processes and gene regulation of cellular metabolism, particularly in the cardiovascular system. Moreover, PPARs have various extra metabolic roles, in circadian rhythms, inflammation and oxidative stress. In this review, we focus mainly on the effects of PPARs on some thermodynamic processes, which can behave either near equilibrium, or far-from-equilibrium. New functions of PPARs are reported in the arrhythmogenic right ventricular cardiomyopathy, a human genetic heart disease. It is now possible to link the genetic desmosomal abnormalitiy to the presence of fat in the right ventricle, partly due to an overexpression of PPAR*γ*. Moreover, PPARs are directly or indirectly involved in cellular oscillatory processes such as the Wnt-b-catenin pathway, circadian rhythms of arterial blood pressure and cardiac frequency and glycolysis metabolic pathway. Dysfunction of clock genes and PPAR*γ* may lead to hyperphagia, obesity, metabolic syndrome, myocardial infarction and sudden cardiac death, In pathological conditions, regulatory processes of the cardiovascular system may bifurcate towards new states, such as those encountered in hypertension, type 2 diabetes, and heart failure. Numerous of these oscillatory mechanisms, organized in time and space, behave far from equilibrium and are “dissipative structures”.

## 1. Introduction

Peroxisome proliferator-activated receptors (PPARs *α*, *β*/*δ*, and *γ*) were initially identified for their key role in metabolic regulatory processes, in the gene regulation of cellular lipid metabolism [1], particularly in heart muscle. PPAR*α* is expressed at relatively abundant levels in the heart and activates numerous genes involved in cellular fatty acid (FA) uptake and oxidation. The functional and biological roles of PPAR*α* include regulation in mitochondrial FA-oxidation, and lipoprotein assembly and transport [1–4]. Numerous papers have reported their complex metabolic interplay in several cardiovascular diseases (cardiac hypertrophy, heart failure) [5–9]. In the cardiovascular system, however, PPARs have various extra metabolic roles, for example, in inflammation, oxidative stress, extracellular matrix remodeling and circadian rhythms [10]. The present paper focuses in particular on: (i) new functions of PPARs in human heart diseases such as arrhythmogenic right ventricular cardiomyopathy (ARVC) [11], a genetic cardiac disease which presents mutations of the desmosomal proteins, and (ii) particular properties of PPARs directly or indirectly involved in cellular oscillatory processes such as the Wnt pathway-*β*-catenin and circadian rhythms of arterial blood pressure and cardiac frequency. Oscillatory phenomena have also been described in the glycolysis metabolic pathway in which PPARs interfere. In their pioneering study, Goldbeter and Lefever [12] have shown that the allosteric phosphofructokinase enzyme generates far-from-equilibrium chemical oscillations [13–15]. Moreover, PPAR dysfunction generates inflammatory phenomena and free radicals for which myosin heads are a new target [16] and the peroxydase enzyme has been found to induce oscillatory processes [17]. Finally, the heart is an open system that exchanges matter and energy with its environment and may operate either near equilibrium or, when myosin crossbridge kinetics become nonlinear in nature [18], far from equilibrium. The lack of PPAR*α*  in the PPAR*α* ^−/−^ mouse model increases the distance from equilibrium in skeletal and cardiac muscles [19]. 

 In this paper, we focus mainly on the effects of PPARs on some thermodynamic processes, which can behave near equilibrium, possibly in stationary state if the thermodynamic flow linearly varies with the thermodynamic force [14]. Other systems lead to oscillatory behavior or unstable states belonging to the family of far-from-equilibrium dissipative structures [13, 20]. They are nonequilibrium thermodynamic systems that operate far from thermodynamic equilibrium and generate order spontaneously by exchanging energy, matter, and/or entropy with their external environment. Dissipative structures include physical processes (whirlpools, cyclones and hurricanes), chemical reactions (Bénard cell convection, the Belousov-Zhabotinsky reaction), biological systems (Turing structures, chirality, circadian rhythms), vegetation self-organization, lasers, and at the most sophisticated level, life itself [14, 21, 22].

## 2. PPARs and ARVC

Arrhythmogenic right ventricular cardiomyopathy (ARVC) is a rare human disease (1/5000) characterized by a unique histological hallmark consisting of a progressive fibrofatty replacement of the right ventricular (RV) myocardium, together with frequent, mostly fibrotic, left ventricular (LV) involvement [23, 24]. This disease is responsible for unexpected syncope or sudden death due to severe ventricular arrhythmias of RV origin, typically occurring under conditions of stress in apparently healthy young people [25]. Diagnosis of the disease is difficult in the early stages and is based on internationally defined criteria [26]. With time, cardiac dysfunction may develop, initially located at the RV and becoming biventricular in at least 20% of cases [27]. At the final stage, heart transplantation remains the only solution available. ARVC is most often a family-related disease with autosomal dominance and 75% male penetrance, while rare autosomal-recessive forms associated with cardiocutaneous syndromes have also been described. Genetic mutations have been identified in about 50% of cases, mostly due to either a single alteration or a combination of two mutations occurring among the five desmosomal proteins so far identified in the ventricular cardiomyocyte: desmoglein 2 (DSG2), desmocollin 2 (DSC2), plakophilin 2 (PKP2), plakoglobin (JUP) and desmoplakin (DSP) [28, 29]. Because ARVC is a specific human disease, a comprehensive approach to the study of the underlying molecular mechanisms has been hampered until recently, when genetically engineered mouse models became available. On the other hand, PPAR abnormalities have been reported in ARVC [11] with an increase in PPAR *γ* and a decrease in PPAR*α*  in the right ventricle. The link between PPAR dysfunction and desmosomal genetic mutations is beginning to be understood via the Wnt/*β*-catenin pathway.

 It has long been shown that *β*-catenin plays a pivotal role during epithelial-mesenchymal transition (EMT), a fundamental phenomenon that characterizes normal embryonic development, tissue regeneration and cancer proliferation (for more details see the paper in [30]). Together with cadherins and *α*-catenin, *β*-catenin is a normal constituent of the zonula adherens, a major cell-to-cell adhesion complex in pavement-like tissues. During EMT, the loss of cadherins due to proteases disrupts the zonula adherens, thus liberating *β*-catenin in the cytoplasm. This molecule then migrates to the cell nucleus where it activates the Wnt/*β*-catenin target genes, initiating the EMT phenomenon: the cells now disrupted from the pavement become more motile and receive mesenchymal properties with high plasticity that enables them to migrate through the basement membrane of the epithelium, thus becoming invasive. The key mechanism in this process is the canonical Wnt pathway. In the absence of a Wnt ligand, the free *β*-catenin becomes phosphorylated in a destruction complex involving axin and other molecules and is finally degraded in the proteasome. Conversely, in the presence of a Wnt ligand, the *β*-catenin degradation complex is inactivated, with recruitment of axin to the plasma membrane, thus stabilizing nonphosphorylated *β*-catenin which translocates to the nucleus; *β*-catenin binds to Tcf/Lef transcription factors. The resulting complex becomes active by displacing Grouchos, leading to activation of numerous target genes. In the myocyte, the net effect is synthesis of contractile protein. Importantly, the canonical Wnt-*β*-catenin-PPAR*γ* system has been shown to determine the molecular switching of osteablastogenesis versus adipogenesis [31]. PPAR*γ* is a prime inducer of adipogenesis that inhibits osteoblastogenesis. Two different pathways switch the cell-fate decision from adipocytes to osteoblasts by suppressing the transactivation function of PPAR*γ*. First, TNF-alpha- or IL-1-induced TAK1/TAB1/NIK signaling cascade attenuates PPAR*γ*-mediated adipogenesis by inhibiting the binding of PPAR*γ* to the DNA response element. Second, the noncanonical Wnt pathway acts through the CaMKII-TAK1/TAB2-NLK signaling cascade. Wnt-5a-induced phosphorylation of NLK triggers synthesis of a complex with the histone methyltransferase SETDB1. This complex represses PPAR*γ* transactivation through histone H3-K9 methylation at the target gene, inhibiting adipogenesis. 

 The  Wnt-*β*-catenin pathway appears to be a similar mechanism for desmosomal abnormalities in ARVC and for some oscillatory processes. A major progress in understanding the molecular mechanisms involved in ARVC has been made by Garcia-Gras et al. [32]. The Wnt/*β*-catenin canonical pathway has been shown to play a role in animal and cell models in which desmosomal proteins are genetically modified. link genetic desmosomal abnormalities to the presence of fat in the right ventricle of ARVC patients, due to dysfunction of PPAR*γ* and *α*. Suppressing DSP expression leads to nuclear translocation of the desmosomal plakoglobin (or *γ*-catenin) by disruption of weakened desmosomes. Garcia-Gras et al. have observed a 2-fold reduction in canonical Wnt/*β*-catenin signaling through Tcf/Lef nuclear transcription factors, a major pathway for contractile protein synthesis. This helps explain the phenotype of ARVC in humans and makes it possible to link genetic desmosomal abnormalities to the presence of fat in the right ventricle of ARVC patients, due to dysfunction of PPAR*γ* and *α*. Other earlier studies have shown that disruption of Wnt/*β*-catenin signaling causes transdifferentiation of myoblasts into adipocytes *in vitro *[33] and leads to activation of PPAR*γ*. Thus, the resulting phenotype in heterozygous transgenic DSP-deficient mice fully recapitulates the human disease, with excess fat and fibrosis in the RV myocardium, increased myocyte apoptosis, decreased contractile proteins accounting for cardiac dysfunction, and ventricular arrhythmias. Garcia-Gras et al. have also postulated that, because plakoglobin (or *γ*-catenin) and *β*-catenin are closely related armadillo proteins, they interact in a competitive and opposite manner upon nuclear localization. Indeed, Ross et al. [33] have long established that plakoglobin-containing Tcf/Lef transcription factor complex binding to DNA is less effective than the corresponding *β*-catenin complex. Moreover, the same authors have shown that Wnt signaling maintains preadipocytes in an undifferentiated state through inhibition of both adipogenic transcription factors, namely CCAAT enhancer binding protein-a (C/EBPa) and PPAR*γ*. The fact that the Wnt-*β*-catenin complex is less effective in the presence of an excess of nuclear plakoglobin would result in less inhibition of PPAR*γ*, thus leading to fat accumulation in the myocardium.

Recently, Lombardi et al. [34] proposed that the net result of the removal of the inhibitory effects of the canonical Wnt signaling is overexpression of bone morphogenic protein 7 (BMP7) and Wnt5b, which have been clearly identified as promoters of adipogenesis, and suppression of the expression of connective tissue growth factor (CTGF), a known inhibitor of adipogenesis induced by PPAR*γ*. Importantly, the authors have also suggested that second heart field progenitor cells, which are the major source of RV cell lineage during embryogenesis, could switch to adipocytes in ARVC, thus accounting for the preferential location of the disease on the right ventricle. On the other hand, Tandri et al. [35] have shown that signal levels for ventricular gap junction protein connexion-43 were diminished in ARVC tissues, thus reinforcing the hypothesis that some other cell-to-cell adhesion systems including gap junctions (and plausibly zonulae adherents) could become fragile and also disrupt as a consequence of desmosome disruption. Gap junction disruption may in turn precipitate intracellular calcium intrusion leading to ventricular premature beats and severe ventricular arrhythmias.

 In a recent study, Djouadi et al. [11] have shown for the first time that RV surviving myocytes from ARVC was characterized by a reduction in expression of PPAR*α*, as compared to control hearts, together with a dramatic activation of the PPAR*γ* pathway, as attested by the increase in PPAR*γ* mRNA and protein. In contrast, the LV exhibited no changes in PPAR*γ* expression, as compared with controls, whereas the expression of PPAR*α* was reduced. Therefore, changes in PPAR signaling may contribute to myocardium fatty accumulation and contractile dysfunction in ARVC. In the same study, the authors also observed heavy neutral lipid accumulation in human ARVC RV myocytes at the boundary between totally fatty transformed myocardium and optically preserved muscle, as also noted earlier by d'Amati et al. [36]. This observation suggests that mature RV myocytes may be directly transformed into mature adipocytes during evolution of the disease. Conversely, LV cardiomyocytes were characterized by heavy fibrosis and little lipid accumulation, consistent with the lack of expression of the PPAR*α* pathway in the ARVC LV myocardium. Finally, it might be postulated that whatever the desmosomal protein invalidated, the net result in the RV would be a nuclear transfer of plakoglobin (*γ*-catenin) with resulting inhibition of Wnt/*β*-catenin signaling and PPAR*γ* overexpression, leading to the unique histological phenotype of fibrofatty replacement and loss of contractile protein expression which summarizes ARVC muscle transformation. Taken as a whole, the Wnt/*β*-catenin pathway appears to be involved in animal and cell models in which desmosomal proteins are genetically modified [32]. This helps explain the phenotype of ARVC in humans and makes it possible to link genetic desmosomal abnormalities to the presence of fat in the right ventricle of ARVC patients, due to dysfunction of PPAR*γ* and *α*  [11].

 Moreover, the Wnt/*β*-catenin pathway through oscillatory processes have recently been described in the somite formation [37]. From a thermodynamic point of view, these oscillatory mechanisms are of major importance [22] because they behave far from equilibrium leading to dissipative structures discovered by Prigogine and colleagues [20, 38].

## 3. PPARs, Cardiovascular System, and Circadian Clock

The cardiovascular system is influenced by numerous extracellular stimuli in a time-of-day-dependent manner [39]. Among the main environmental fluctuations are light, temperature, nutrients and humidity. The myocardial metabolism also exhibits diurnal variations. An intramyocardial circadian clock, which represents a cell-autonomous molecular mechanism, regulates the myocardial metabolism either directly (via triglyceride and glycogen metabolism) or indirectly (via modulation of the myocardium function in response to changes in workload, insulin secretion, and fatty acid supply). This intramyocardial circadian clock makes it possible to anticipate changes in environmental stimuli, that is, changes in workload or feeding status prior to their onset. Disruption of the circadian clock may impair the synchronization between the myocardium and its environment and may result in pathogenesis such as observed in diabetes mellitus. Generally, environmental factors fluctuate in a predictable manner (light/dark cycles), others are less predictable (feeding/fasting cycles). They also depend either on the time of day (i.e., circadian) or year (i.e., seasonal). The primary timekeeping mechanism [40] is the intracellular circadian clock [41–43]. Circadian clocks are cell-autonomous molecular mechanisms and are transcriptionally-based positive and negative feedback loops with a free running period of about 24 hours. Mammalian circadian clock mechanisms involve two critical transcription factors, CLOCK and BMAL1 [44, 45]. CLOCK/BMAL1 heterodimerization leads to formation of both positive (BMAL1) and negative (PER1/2/3, CRY1/2, rev-erb-alpha, DEC1/2) loops, the latter repressing the transcriptional activity of the CLOCK/BMAL1 heterodimer. The CLOCK/BMAL1 heterodimer also influences several clock output genes, so that the circadian clock may alter the cellular function over the course of the day. Clock genes themselves link the core molecular clock and metabolic regulatory networks. The nuclear receptor and core clock component rev-erb-alpha behaves as a gatekeeper, coordinating the circadian metabolic response in a timely manner [46].

 The circadian clock mechanism allows the cell to perceive the time of day and facilitates cellular responses to environmental stimuli in both a rapid and temporally appropriate manner. This may confer a selective advantage of anticipation. Circadian rhythmicities are involved in cardiovascular physiology (heart rate, cardiac output, arterial blood pressure) and pathophysiology (arrhythmias) [47, 48]. Sleep/wake and feeding/fasting cycles are two major physiologically environmental circadian cycles influencing the myocardial function in its property of anticipating metabolic processes. Contractile function is closely related to myocardial metabolism and limitation of ATP synthesis may impair myocardial contractile function and survival of patients [49, 50]. The myocardial metabolism is able to adapt rapidly to environmental stimuli both during physiological situations such as developmental transitions from fetal to adult state and during pathophysiological conditions, such as heart failure [51–53]. Importantly, over the 24-hour period, sleep/wake and feeding/fasting cycles markedly influence myocardium function. Periods of wakefulness are associated with an increase in physical activity, heart rate, cardiac output and food consumption [54, 55]. The circadian clock within the cardiomyocyte mediates diurnal variations in the responsiveness of the heart to increased workload, according to contractile function and metabolic flux levels [56]. It has been found that there is a diurnal variation in the transcriptional response of the heart to fatty acids, which occurs in a cardiomyocyte circadian clock-dependent manner [57, 58]. The heart responds to fatty acid variations by activating PPARs [59]. PPAR*α* can mediate diurnal variations in the responsiveness of the heart to both fatty acids and specific PPAR*α* agonism (WY-14 643) [57]. The PPAR*α*  gene is a circadian clock-regulated gene in the liver [60]. In the normal heart, PPAR alpha mRNA exhibits only weak circadian oscillations. This raises the question of the importance of the role of the PPAR*α*  gene in the cardiomyocyte circadian clock [47, 56, 57]. 

 Recently, it has been demonstrated that vascular PPAR*γ* is a peripheral regulator of cardiovascular rhythms which controls circadian variations in blood pressure and heart rate through BMAL1 [61]. PPAR*γ*  appears to be a key component of the vascular clock. In mice with vascular PPAR*γ* deletion (Tie2-Cre/flox and SM22 Cre/flox mice), the circadian fluctuations in heart rate and blood pressure are reduced. Thus, circadian rhythms of blood pressure and heart rate appear, at least partly, to be regulated by a PPAR*γ*-dependent mechanism which is peripheral and intrinsic to blood vessels. Pioglitazone, a PPAR*γ* activator, shifts the circadian rhythm of blood pressure from nondipper to dipper type 2 diabetes patients [62]. In PPAR*γ* mutant mice, the impairment of cardiovascular rhythmicity parallels the diurnal variations in urinary excretion of norepinephrine and epinephrine (estimates of the overall sympathetic tone), which are suppressed [61], similarly to that observed in BMAL1^− /−^  mice [63] and in Cry-deficient mice lacking a biological clock [64]. As discussed by Wang and colleagues, several evidences show that PPAR*γ* acts as a direct regulator of BMAL1 in blood vessels [61]; (i) a vascular PPAR*γ* oscillation precedes BMAL1; (ii) in blood vessels, PPAR*γ* deficiency induces a blockade of rhythmicity and baseline BMAL1 expression in the aorta; (iii) a direct interaction between PPAR*γ*  and BMAL1 has been demonstrated by means of a ChIP assay; (iv) rosiglitasone induces aortic expression of BMAL1; (v) in vascular cell, BMAL1 promotor is responsive to rosiglitasone and this is abolished by mutagenesis at the PPRE site level. Changes in feeding time or in photoperiod reset the phase of rhythmic expression of vascular PPAR*γ*. This supports a role for PPAR*γ* as a component of the peripheral clock. The major role of PPAR*γ* in glucose and lipid metabolism [65, 66] and the discovery of the circadian properties of PPAR*γ* [61] may confer to PPAR*γ* a coordinated function between metabolism and circadian rhythms. Moreover, PPAR*α* directly interacts with BMAL1 and regulates the peripheral hepatic oscillator [67]. Overall, these data suggest that the peripheral clock BMAL1 and different PPAR subtypes interfere in a complex manner to regulate the metabolic function of glucose and lipid and the cardiovascular circadian rhythms of blood pressure and heart rate. Pathophysiological consequences of circadian dysfunctions may be important. In normal humans, a sharp rise in blood pressure occurs before awaking and peak values appear around midmorning. Sudden cardiac death, myocardial infarction and stroke often occur at the early morning surge in blood pressure [68, 69]. BMAL1 and CLOCK dysfunction leads to hyperphagia, obesity and metabolic syndrome including hyperlipidemia, hepatic steatosis, hyperleptinemia and hyperglycemia [70–72].

 Circadian rhythms originate from the negative feedback which is exerted by a protein on the expression of its gene [73, 74]. A. Goldbeter has proposed molecular models for circadian oscillations of the period (PER) protein which behaves as a transcriptional regulator modulating several genes besides PER its own gene. For appropriate parameter values of the molecular model, the steady state becomes unstable and limit-cycle oscillations appear. These far-from-equilibrium systems have been described as dissipative structures [20]. As previously reported, a segmentation clock has been demonstrated for genes involved in Wnt, Notch and Fibroblast Growth Factor (FGF) signaling pathways [37]. The segmentation clock controls the periodic formation of somites in vertebrate embryos and represents a complex example of biological rhythm. This oscillatory phenomenon combines temporal and spatial self-organization [75, 76]. Thus, the Wnt/*β* catenin pathway interferes in oscillatory biological processes and modulates the PPARs, themselves subject to rhythmic oscillations [60]. Such nonlinear systems favor the occurrence of instabilities and thermodynamically exhibit far-from-equilibrium behavior.

## 4. PPARs, Pyruvate Dehydrogenase, Phosphofructokinase, Glycolytic Oscillations, and Dissipative Structures

PPARs play a key role in the glucose metabolic pathway [77, 78]. Over expression of the PPAR*α* isoform in skeletal and cardiac muscles of mice drives diminished glucose transporter gene expression and glucose uptake into these insulin target tissues [79]. In heart muscle perfused with FA and ketone bodies, the glycolytic rate is decreased. The impairment of glucose degradation occurs at four key steps [80], that is, membrane transport of glucose, glucose phosphorylation, phosphofructokinase and pyruvate dehydrogenase complex (PDC), metabolic conditions where PPAR*α* is modified. Moreover, in type 2 diabetes, there is an increased expression of PPAR*α* and its target genes involved in fatty acid metabolism in skeletal muscle of Zucker Diabetic Fatty (ZDF) rats. In contrast, the mRNA levels of genes involved in glucose transport and utilization (GLUT4 and phosphofructokinase) have been shown to be decreased, whereas the expression of pyruvate dehydrogenase kinase-4 (PDK-4), which suppresses glucose oxidation, is increased [81]. The shift from glucose to fatty acids as the source of energy in skeletal muscle of ZDF rats is accompanied by a decrease of subunit 1 of complex I and subunit II of complex IV, two genes of the electronic transport chain encoded by mtDNA, and a decrease in transcript levels of PPAR*γ* Coactivator 1 (PGC-1). Inhibition of PDC occurs by activation of the PDKs after an increase in the mitochondrial acetyl-CoA/CoA and NADH/NAD^+^ ratios generated by high fatty acid oxidation rates. In the heart, PDK-4 is a target gene for upregulation by PPAR*α* which forces FA oxidation by blocking pyruvate oxidation [79].

Glycolysis reactions involve glucose, ADP, NAD, pyruvate, ATP, and NADH, according to the following overall reaction:

(1) $$\begin{array}{cc} & \text{Glucose}+2\text{ADP}+2\text{Pi}+2\text{NAD}\\ & \;\Leftrightarrow 2\;\text{Pyruvate}+2\text{ATP}+2\text{NADH}. \end{array}$$

A reaction of the process is autocatalyzed by phosphofructokinase (PFK), responsible for glycolytic oscillations. PFK, an allosteric enzyme, can lead to instabilities beyond which a new state can be organized in time and in space [82, 83]. This open monosubstrate enzyme is activated by the product of the reaction. Positive feedback is responsible for periodic behavior and glycolytic oscillations are due to activation of PFK by its reaction product. These oscillations were obtained in a two-variable model for the product-activated PFK reaction responsible for glycolytic oscillations [84, 85]. Limit-cycle oscillations represent a model of nonequilibrium self-organization. PPAR*α* interferes in PDK, PDC and PFK activities and the cellular regulation of these four proteins is influenced by circadian rhythms. These far-from-equilibrium oscillatory mechanisms enter the field of dissipative structures initially described by Illia Prigogine [12, 20].

## 5. PPAR*α* Deficiency and Cardiac Performance

The functional and biological roles of PPAR*α* in cardiac muscle have been extensively investigated through the PPAR*α* ^−/−^ mouse model [2, 4, 86]. Although PPAR*α* ^−/−^ mice have a normal life span, they develop progressive cardiac fibrosis with abnormal myofibrils and mitochondria [4]. Cardiac abnormalities have been reported in PPAR*α* ^−/−^ mice [87, 88]. Histological studies revealed significant cardiomyocyte hypertrophy in PPAR*α* ^−/−^ mice [16]. *Ex vivo* left ventricular papillary muscle exhibits reduced shortening velocity and isometric tension. The reduced *in vitro* myosin-based velocity in PPAR*α* ^−/−^ heart strongly suggests that pathological processes that directly affects the myosin molecule itself are involved in cardiac dysfunction in PPAR*α* ^−/−^ mice [16, 89–91]. Echocardiographic left ventricular fractional shortening is also reduced in PPAR*α* ^−/−^ mice [16]. From a thermodynamic point of view, myosin II molecular motors from PPAR*α* ^−/−^ mouse diaphragm muscle behave in a near-equilibrium manner and in a stationary state [19], although those from heart muscle might operate farther from equilibrium than does diaphragm myosin II [16]. This would be due to high values of crossbridge kinetics and low values of crossbridge efficiency in mouse heart muscle [92]. The lack of PPAR*α* induces an increase of the distance from equilibrium according to the type of myosin II molecular motors.

## 6. PPAR*α*, Redox Balance and Heart: Myosin II Molecular Motor, a Target for Oxidative Stress

A balance between oxidants and antioxidant defenses is required to maintain the homeostasis of cardiac function [93–97]. In a physiological range, the antioxidative response to a moderate increase in ROS may be sufficient to reset the balance between ROS production and ROS scavenging capacity [98, 99] and the relation between ROS concentration and muscle performance is a bell-shaped relationship. Thus redox homeostasis and muscle function can be maintained in a near-equilibrium stationary thermodynamic state [14, 19] or quasi-stable state [100]. In the PPAR*α* ^−/−^ mouse model, the redox system is subjected to dramatic and/or long-lasting perturbations, and cardiac dysfunction appears with direct impairment of the myosin II heads [16]. Depletion of antioxidant enzymes such as superoxide dismutase (SOD), catalase, and glutathione peroxidase (GPX) or overproduction of reactive oxygen species (ROS) or reactive nitrogen species (RNS), may induce oxidative stress and cause cardiac functional disorders [97, 101]. Importantly, it has been shown that oscillations may be induced by peroxidase [17], and the waveform of the oscillations changes when the concentration of peroxidase is varied. There is strong evidence that activation of PPAR*α* is necessary to prevent cellular oxidative damage that may occur during physiological cellular metabolism or under conditions of inflammation and oxidative stress, probably through repressing nuclear factor-*κ*B signaling and reducing inflammatory cytokine production [102–104]. Therefore, chronic deactivation of the PPAR*α* signaling pathway may upset normal equilibrium between oxidant production and antioxidant defenses and contribute to cardiac damage. SODs act as a first line of defense against oxidative stress by catalyzing the dismutation of superoxide anions to hydrogen peroxide (H_2_O_2_). Subsequently, H_2_O_2_ is reduced to H_2_O and O_2_ by peroxidase or catalase [105]. Regulation of MnSOD is considered to play a crucial role in cardiac oxidative stress [106, 107]. There is a decrease in MnSOD expression in PPAR*α* ^−/−^ hearts compared with WT, associated with an even more pronounced decrease in enzymatic activity [16, 106, 107]. This increases the level of superoxide, which in turn may react with the NO to form the powerful oxidant peroxynitrite and induce protein nitration. 

 In heart, myofibrillar [96, 108] as well as mitochondrial [109] proteins are major targets for oxidative stress-derived effects. Changes in protein structure have been shown to modulate cardiac function [93, 97]. Therefore, oxidative and/or nitrosative modifications of contractile proteins may contribute to cardiac dysfunction in PPAR*α* ^−/−^ mice. For example, protein tyrosine-nitration has been shown to be involved in several cardiomyopathies [93, 97, 110]. In PPAR*α* ^−/−^ heart in particular, myosin heavy chain represents a major target for protein tyrosine nitration [16]. Alterations in the conformation of myosin II molecular motors by nitration account for the reduced myosin-based velocity observed in PPAR*α* ^−/−^ heart and consequently account for the decreased maximum unloaded shortening velocity and total tension of left ventricular papillary muscles. Moreover, PPAR*α* ^−/−^ hearts exhibit a substantial increase in HNE-protein adducts, thus attesting to increased lipid peroxidation, which contributes to contractile alterations [105]. Peroxynitrite may also impair myocardial contractility via other mechanisms including activation of matrix metalloproteinases, nuclear enzyme poly (ADP-ribose) polymerase, induction of apoptosis in myocytes, impairment of mitochondrial function, or abnormalities in calcium cycling [110]. Potential sources of increased production of superoxide in PPAR*α* ^−/−^ heart include activation of various enzyme complexes such as NADPH oxidase and xanthine oxidase. Increased myocardial NO may result from different sources, such as increased myocardial iNOS activity [110]. Generation of peroxynitrite can trigger posttranslational modifications of proteins such as S-glutathiolation or S-nitrosylation as well as severe oxidative injury leading to the induction of cell death [110].

## 7. Conclusion

Taken as a whole, this paper shows how PPARs in the cardiovascular system are involved in physiological and pathological metabolic processes and circadian rhythms, often involving oscillatory systems modulated by complex gene activities. These oscillatory systems behave far from equilibrium and their concerted activities make it possible to adapt the glucose/free fatty acids metabolism to the cardiovascular function, according to circadian rhythms of blood pressure and heart rate [61]. In pathological conditions, these regulatory processes of the cardiovascular system may bifurcate towards new states, such as those encountered in hypertension, type 2 diabetes, cardiac hypertrophy, myocardial infarction, and heart failure. PPAR*α* gene regulatory pathway activity is downregulated in hypertrophied heart [111] and in the human failing heart [111–114]. It is unclear whether the metabolic shift, with increased reliance on glucose metabolism rather than fatty acid oxidation, induces a protective response allowing the heart to maintain contractile function or is the initial step leading to progressive deterioration of contractile function [111, 115]. Dysfunction of BMAL1, CLOCK and PPAR*γ* may lead to obesity and metabolic syndrome [70–72]. PPARs are involved in several oscillatory systems (phosphofructokinase, circadian rhythms, Wnt/*β*-catenin pathway, etc.). The Wnt-*β*-catenin pathway, an oscillatory system involved in somite formation, seems to help explain the pathophysiology of ARVC in which PPAR*α* and *γ* are disturbed. Prigogine and colleagues have shown that such organized structures in time and space behave far from equilibrium and are “dissipative structures” [116–120].
